# Postabortion Family Planning and Associated Factors Among Women Attending Abortion Service in Dire Dawa Town Health Facilities, Eastern Ethiopia

**DOI:** 10.3389/frph.2022.860514

**Published:** 2022-06-16

**Authors:** Venus Shewangizaw Motuma, Tesfaye Assebe Yadeta, Addisu Alemu, Mohammed Yuya, Bajrond Eshetu, Bikila Balis, Miressa Bekana, Bedasa Taye Merga, Lemessa Oljira

**Affiliations:** ^1^CARE International Ethiopia, Nutrition Project, Harar, Ethiopia; ^2^School of Nursing and Midwifery, College of Health and Medical Science, Haramaya University, Harar, Ethiopia; ^3^School of Public Health, College of Health and Medical Science, Haramaya University, Harar, Ethiopia; ^4^School of Medicine, College of Health and Medical Science, Haramaya University, Harar, Ethiopia

**Keywords:** women, postabortion, family planning, Dire Dawa, Ethiopia

## Abstract

**Background:**

Postabortion family planning is a part of comprehensive package of postabortion care. However, it did not receive due attention to break the cycle of repeated abortion, unintended pregnancies, and abortion-related maternal morbidity and mortality. Therefore, this study aimed to determine the utilization of postabortion family planning and associated factors among women attending abortion service in Dire Dawa health facilities, Eastern Ethiopia.

**Methods:**

A facility-based cross-sectional study design was employed among 483 clients who sought abortion service in Dire Dawa from 15 May to 30 June 2020. A structured interviewer-administered questionnaire was used for data collection. The collected data were entered into EpiData version 3.2 and exported to SPSS version 22 for analysis. The multivariate logistic regression models were fitted to identify factors associated with utilization of postabortion family planning. Adjusted odds ratios (AORs) along with 95% CI were estimated to measure the strength of the association and statistical association was declared statistical at a *p*-value < 0.05.

**Results:**

More than three-fourths (77.8%) [95% CI: (73.4–81.6%)] of respondents were utilized postabortion family planning methods. Respondents with age 15–24 years [AOR: 5.59, (95% CI: (1.5, 19.7)], attended postsecondary [AOR: 7.6, (95% CI: (2.7, 21.2)], single marital status [AOR: 11.1, (95% CI: (4.6, 26.5)], a monthly income 500–1,000 birr [AOR: 7.9, (95% CI: (3.2, 19.4)], parity ≥ 5 [AOR: 0.41, (95% CI: (0.18, 0.92)], desire of current pregnancy [AOR: 4.2, (95% CI: (1.9, 9.3)], and ever used family planning [AOR: 4.4, (95% CI: (2.2, 8.9)] were major factors significantly associated with postabortion family planning utilization.

**Conclusion:**

In this study, more than three-fourths of respondents utilize postabortion family planning. Most of the factors associated with postabortion family planning were modifiable. Therefore, policymakers and health planners need to integrate with comprehensive abortion care to improve the utilization of postabortion family planning.

## Introduction

Abortion is the top five obstetrics cause for maternal death worldwide (1, 2). Every year, a total of 121 million women face unintended pregnancy and 73.3 million women (61%) end with an induced abortion (3). Ethiopia is one of the sub-Saharan African countries highly hit with abortion, 382,500 every year (4). Abortion has a direct contribution of 810 maternal deaths (2, 5). Also, in sub-Saharan Africa, unsafe abortion disproportionately affecting women, 520 deaths per 100,000 women every year (3).

Almost every abortion, death and disability could be prevented through sexuality education, use of effective family planning, provision of safe, legal-induced abortion, and timely care for complications (5, 6). Family planning is the best method in preventing unintended pregnancy and related complications. However, most of the time, postabortion family planning services were ignored by health providers and clients (7). In Ethiopia, only 41% of currently married women were using family planning (8).

The WHO endorses postabortive women to use family planning methods at least 6 months before subsequent pregnant (9). Family planning is a part of comprehensive package of postabortion care (10) and it is an important strategy to reduce maternal morbidity and mortality (11) by breaking the cycle of repeated abortion and unintended pregnancy (12). However, national-level evidences revealed that there is a significant disproportion between safe abortion and postabortion family planning (PAFP) utilization (13).

Likewise, evidences in Ethiopia have shown that 65–79% women utilize postabortion family planning (14, 15) The utilization of PAFP was affected by different socioeconomics, sociodemographic characteristics, sexual behaviors, and knowledge level of the clients (14, 16). In Ethiopia, modern methods of family planning have been advocated for the last 50 years to prevent unwanted pregnancy (17). Nevertheless, there is a large gap between actual fertility and women's average preferred family size in Ethiopia (8). Also, unmet need for family planning is 22% among married women (18).

Evidence is needed to address the reproductive health need of a woman seeking abortion service, to design and implement the services. The high prevalence of unmet need for PAFP among women undergone induced abortion needs update information. Moreover, identifying factors associated with postabortion family planning may help to devise contextualized strategies that could improve the utilization of postabortion family planning. Therefore, this study aimed to assess utilization of postabortion family planning and associated factors among women attending abortion service in Dire Dawa, Eastern Ethiopia.

## Methods and Materials

### Study Area, Design, and Period

A facility-based cross-sectional study was conducted in Dire Dawa. Dire Dawa is one of the administrative cities of Ethiopia and covers an area of 1,977 km^2^. The city is located in the Eastern Ethiopia at a distance of 515 km from Addis Ababa. It has a total population of 341,834 (171,461 males and 170,373 females). More than two-thirds, 233,224 (68.23%) of the populations are urban residents (18). Health service coverage of the city is 95% and the city has two public and four private hospitals, 15 health centers, 34 health posts, and 53 private clinics (19). Abortion is legalized in 6 hospitals and 15 health centers. This study was conducted in 12 health facilities found in Dire Dawa town and from 15 May to 30 June 2020.

### Source Population, Study Population, and Eligibility Criteria

All the clients who sought abortion services were included in this study. A sample of clients who have undergone abortion care services was the study population. Medically eligible clients to any family planning methods and clients who gave volunteer-based consent were included in this study. Clients who are not medically eligible for any type of family planning methods were excluded.

### Sample Size Determinations

The sample size was determined based on predictors of postabortion family planning counseling and using statistical Epi Info™ 7 StatCalc computer software program using 95% CI with *Z* = 1.96, the proportion of postabortion family planning among those ever used contraceptive (75.2%) and never used contraceptive (62.6%) (20) and 80% power, 10% nonresponse (*n* = 483). Clients who were not medically eligible to any type of family planning methods were excluded. The sample from each hospital and health center was allocated proportionally to the number of clients in each institution.

### Sampling Techniques

All the 12 health facilities (two nongovernmental clinics—which were ran by Marie Stops International and Ethiopian Family Guidance Association, one public referral hospital, two private hospitals, five public health centers, and two special clinics providing obstetrics and gynecological service) that giving family planning services in Dire Dawa town were included in this study. Based on a preliminary assessment of the past 3 months, using proportional allocation technique to size, sample size was calculated for each health institution delivering the service and finally, a total of 483 participants were selected based on their allocation. Sabian Primary Hospital, Bilal Private Hospital, three primary healthcare units (PHCUs), and one private clinic were excluded from this study due to an availability of abortion cases and serving as coronavirus disease 2019 (COVID-19) center during the assessment of 3-month preliminary data. All of the women who seek for the abortion service in health facilities were included consecutively until the required sample was fulfilled and the participants were reached through an exit interview after the women get an abortion service at selected health facilities and the interview was conducted in a private place in the facility.

### Data Collection Tools

The questionnaires were adapted from different literatures (15, 21). It had three parts: sociodemographic characteristics, reproductive-related characteristics, and utilization of postabortion family planning. The questionnaire was prepared in English, translated to local languages (Amharic, Afaan Oromoo, and Somali language) by those who were proficient of the languages, and again translated back into English to check the consistency of the questionnaire.

### Quality Control and Data Collection Producers

Five percent (24) of the questionnaire was pretested in other health facilities that were providing PAFP services. Then, ambiguous questions were corrected and rephrased before actual data collection. The data collectors and supervisors were trained for 2 days about the objectives of this study, COVID-19 prevention, content of tools, and technique of interview before data collection hurled. After all, clients who were consented to participate in pretested and structured interviewer-administered questionnaires were used to collect the data in the private room in the facilities. The data were collected by 12 trained diploma Nurses and two BSc Nurses. Supervisors were monitored the whole data collection process closely and regularly. All the eligible participants were conveniently included in this study till the sample size was fulfilled. Moreover, participants were informed about the study title, purpose, procedure, risk and benefits, rights, and confidentiality of this study. The next legible mother has replaced those women who did not fulfill the inclusion criteria.

### Data Processing and Analysis

The data were coded, entered into Epi data version 3.2, and cleaned and exported to SPSS version 22 for analysis. Multicollinearity assumptions were checked by variance inflation factor (VIF) and variables with VIF 12 larger than 10 were omitted from the final model. Fitness of the model was tested by the Hosmer–Lemeshow goodness of fit test (*p*-value = 0.089). Descriptive statistics were used to present frequency distributions. The bivariate analysis was employed to identify factors associated with utilization of postabortion family planning methods. Variables with *P*-value <0.25 during the bivariate analysis were entered into the multivariate logistic regression models to control for all the possible confounders to identify factors associated with utilization of postabortion family planning methods. Adjusted odds ratios (AORs) along with 95% CI were estimated to measure the strength of the association. Statistical association was declared at a *p*-value <0.05.

### Operational Definition

#### Postabortion Family Planning Utilization

It is the initiation and use of modern contraceptive methods at the time of management of abortion or before fertility returns after the abortion (22).

**Abortion** is defined as the termination of a pregnancy before 28 weeks of gestation(23).

## Results

### Sociodemographic Characteristics

A total of 473 participants were interviewed with a response rate of 97.9%. Around three-fourths, 341 (72.1%) respondents were urban residents. One-thirds, 159 (33.6%) and 175 (33.2%) respondents were found in age range of 25–34 and 15–24 years, respectively. Majority of 224 (47.5%) participants never married. Regarding education and occupation, 234 (49.4%) participants were attended postsecondary education and 217 (45.9) participants were involved in private occupation, respectively (Table 1).

**Table 1:** Sociodemographic characteristics of women who came for abortion care services in Dire Dawa town, Eastern Ethiopia, 2020 (*n* = 473).

**Characteristics**	**Categories**	**Frequency**	**Percent (%)**
Age of women	15–24	157	33.2
	25–34	159	33.6
	35–44	113	23.8
	≥45	44	9.4
Residence of women	Urban	341	72.1
	Rural	132	27.9
Occupation status of women	Government Private	105 217	22.2 45.9
	House Wife	74	15.6
	Daily Labor	68	14.4
	Students	9	1.9
Education level of women	No Formal education	73	15.4
	Primary (1–8)	59	12.4
	Secondary (9–12)	107	22.6
	Post-secondary	234	49.4
Religion of women	Muslim	276	58.4
	Orthodox	158	33.0
	Protestant	41	8.7
Marital Status of women	Married	130	27.5
	Single	224	47.5
	Divorced	39	8.2
	Widowed	13	2.7
	Separated	34	7.2
Average monthly household income	<500birr 500–1000birr	77 57	16.3 12.1
	>1000birr	339	71.7

More than half, 265 (56%) respondents were grand para and 247 (52.5%) respondents had ever gave birth. From previous pregnancy, 191 (40.4%) respondents end up with abortion and 180 (94.2%) respondents had one time abortion. Majority of 90 (47.1%) of the abortion take place in government health facilities followed by private clinic 79 (40.4%) and 114 (59.7%) of respondents terminated their pregnancy by medical termination (Table 2).

**Table 2:** Reproductive characteristics of women who came for abortion care services in Dire Dawa town, Eastern Ethiopia, 2020 (*n* = 473).

**Characteristics**	**Category**	**Frequency**	**Percent (%)**
Gravidity	1	63	13.3
	2–4	145	30.7
	≥5	265	56.0
Parity	Yes	247	52.5
	No	226	47.8
Number of alive children	0	211	44.6
	2–4	195	41.2
	≥4	67	14.2
Previous history of abortion	Yes	191	40.4
	No	282	59.6
Type of abortion (*n* = 191)	Induced	134	70.2
	Spontaneous	57	29.8
Number of abortion (*n* = 191)	1	180	94.2
	≥2	11	5.8
Place of abortion service (*n* = 191)	Public health facilities	90	47.1
	Private clinic	79	40.4
	Others^*^	22	11.5
Desire of current pregnancy	Yes	91	19.2
	No	382	80.8
Desire to have children in the future	Yes	375	79.3
	No	98	20.7
Method used to terminate current pregnancy	Medical	114	59.7
	Surgical	51	26.7
	Mixed method	26	13.6
Reasons for termination	Rape	36	7.6
	Income	76	16.0
	Maternal indication	60	12.7
	Fetal deformity	23	4.9
	Spontaneous	125	26.4
	Unwanted	153	32.3
Duration of current pregnancy	<12 Weeks	367	77.6
	>12 Weeks	106	22.4

* *Private pharmacy, home delivery*.

### Utilization of Postabortion Family Planning

More than seven out of ten, 341 (72.1%) respondents ever used at least one method of family planning, where 165 (38.2%) respondents were used injection, 89 (20.6%) respondents were used oral contraceptives, 47 (10.9) respondents were used implants, and 19 (4.9%) respondents were used an intrauterine contraceptive device (IUCD), respectively. Nearly two-thirds, 306 (64.7%) respondents were not informed when to have pregnancy.

More than three-fourths, 368 (77.8%) respondents were utilized PAFPs, where 193 (52.4%) respondents were used injection, 89 (24.2%) respondents were used implants, 51 (13.8%) respondents were used oral contraceptives, and 23 (6.3%) respondents were used IUCD, respectively. The most common reasons for not utilizing the PAFP were had no reason 42 (40.0%) and child desire 17 (16.2%). Fifth-seventh, 260 (70.7%) respondents decide contraceptive methods they want to take by themselves (Table 3).

**Table 3:** Utilization of postabortion family planning of women who came for abortion care service in Dire Dawa town, Eastern Ethiopia, 2020 (*n* = 473).

**Characteristics**	**Category**	**Frequency**	**Percent**
Ever used family planning methods	Yes	341	72.1
	No	132	27.9
Type of methods ever used	Oral contraceptives	89	20.6
	Injectable	165	38.2
	Condom	21	4.4
	Implant	47	10.9
	IUD	19	4.9
Family planning method during the index of pregnancy	Yes	43	9.1
	No	430	90.9
Type of methods used during the index of pregnancy	Oral contraceptive	24	55.8
	Injectable	12	27.9
	Condom	7	16.3
Informed when to have pregnancy	Yes	167	35.3
	No	306	64.7
Counseling on post-abortion family planning	Yes	369	78.0
	No	104	22.0
Post-abortion utilization of any family planning methods	Yes	368	77.8
	No	105	22.2
Type of family planning received after abortion	Oral contraceptive	51	13.8
	Injectable	193	52.4
	Condom	12	3.3
	Implant	89	24.2
	IUD	23	6.3
Reasons for not using contraceptives	Failed methods	2	1.9
	Side effect	8	7.6
	Husband pressure	16	14.9
	Want a child	17	16.2
	Husband pressure	9	8.5
	Religious ground	11	10.5
	No reason	42	40.0
Decision to receive contraceptives	Myself	260	70.7
	My husband	27	7.3
	Family member	7	1.9
	Health professionals	74	20.2

### Factors Associated With Utilization of Postabortion Family Planning

In the bivariate logistic regression, age, residence, educational level, marital status, household monthly income, parity, history of abortion, desire of current pregnancy, ever used any family planning methods before abortion, and counseled on postabortion family planning were factors significantly associated with utilization of postabortion family planning methods.

However, in the multiple logistic regression, respondents with 15–24 years of age were six times more likely utilized postabortion family planning [AOR: 5.59, (95% CI: (1.5, 19.7)] and respondents who have attended postsecondary were almost eight times more likely utilized postabortion family planning [AOR: 7.6, (95% CI: (2.7, 21.2)]. Similarly, respondents with single marital status were 11 to 16 times [AOR: 11.1, (95% CI: (4.6, 26.5)] and divorced were 16 times [AOR: 16.4, (95% CI: (1.6, 16.6)] more likely utilized postabortion family planning methods. Respondents who had monthly income of 500–1,000 birr five times [AOR: 5.3, (95% CI: (1.5, 18.1)] and > 1,000 birr eight times [AOR: 7.9, (95% CI: (3.2, 19.4)] more likely utilized postabortion family planning, respectively.

Respondents who were not desire of current pregnancy and who ever used any family planning method before abortion were four times more likely utilized postabortion family planning, respectively [AOR: 4.2, (95% CI: 1.9, 9.3)] and [AOR: 4.4, (95% CI: (2.2, 8.9)]. Similarly, counseling on family planning was six times more likely used postabortion family planning methods. Respondents who had 2–4 [AOR: 0.09, (95% CI: (0.23, 0.36)] and ≥ 5 [AOR: 0.41, (95% CI: (0.18, 0.92)] parity were less likely utilized postabortion family planning methods, respectively (Table 4).

**Table 4:** Factors associated with utilization of postabortion family planning among women attending abortion care service in Dire Dawa town, Eastern Ethiopia, 2020.

		**Utilization of PAFP**	
**Variables**	**Category**	**Yes (%)**	**No (%)**	**COR(95 % CI)**	**AOR(95 % CI)**
Age	15–24	139 (88.5)	18 (11.5)	**3.8 (1.6**–**8.03)**	**5.59 (1.5**–**19.7)**
	25–34	124 (80.0)	35 (20.0)	1.6 (0.79–3.4)	1.84 (0.57–5.88)
	35–44	75 (66.3)	38 (33.7)	0.92 (0.4–1.9)	1.9 (0.37–3.85)
	45+	30 (68.0)	14 (32)	1	1
Level of education	No Education	35 (47.9)	38 (52.1)	1	1
	Primary	41 (69.4)	18 (30.6)	2.4 (1.2–5)	2.9 (0.91–9.3)
	Secondary	80 (74.7)	27 (25.3)	3.2 (1.7–6)	2.5 (0.91– 6.9)
	Post-secondary	212 (90.5)	22 (9.5)	**10.4 (5.5**–**19.7)**	**7.6 (2.7**–**21.2)**
Marital status	Married	74 (57)	56 (43)	1	1
	Single	203 (90.6)	21(9.4)	**7.3 (4.1**–**12.9)**	**11.1 (4.6**–**26.5)**
	Divorced	35 (89.7)	4 (10.3)	**6.62 (2.2-19.7)**	**16.4 (1.6**–**16.6)**
	Widowed	31(67.3)	15 (32.7)	1.56 (0.77–3.17)	2.24 (0.75–6.6)
	Separated	25 (73.5)	9 (26.5)	2.1 (0.91–4.8)	2.4 (0.74–7.8)
Monthly income	<500	39 (50.6)	38 (49.4)	1	1
	500–1000	45 (78.9)	12 (21.1)	**3.65 (1.6**–**7.9)**	**5.3 (1.5**–**18.1)**
	>1000	284 (83.7)	55 (16.3)	**5.03 (2.9**–**7.9)**	**7.9 (3.2**–**19.4)**
Residence	Urban	279 (88)	62 (18)	2.17 (1.3–3.4)	1.2 (0.57–2.6)
	Rural	89 (67.5)	43 (32.5)	1	1
Parity	1	29 (46)	34 (54)	1	1
	2-4	109 (75)	36 (25)	**3.5 (1.9**–**6.6)**	**0.09 (0.23**–**0.36)**
	≥5	230 (86.7)	35 (13.3)	**7.7 (4.2-14.1)**	**0.41 (0.18**–**0.92)**
History of abortion	Yes	162 (84.8)	29 (15.2)	2.06 (1.2–3.3)	1.4 (0.07–2.9)
	No	206 (73)	76 (27)	1	1
Desire of current pregnancy	Yes	53 (58.2)	38 (41.8)	1	1
	No	67 (17.5)	315 (82.5)	**3.3 (2.05**–**5.5)**	**4.2 (1.9**–**9.3)**
Ever used methods	Yes	300 (88)	41 (12)	**6.88(4.3**–**1.04)**	**4.4 (2.2**–**8.9)**
	No	68 (51.5)	64 (18.5)	1	1
Counseling on family planning	Yes	59 (16)	310 (84)	**4.16 (2.5**–**6.7)**	**5.7 (2.5**–**12.8)**
	No	46 (44.2)	58 (55.8)	1	1

## Discussion

Family planning utilization especially, postabortion family planning methods utilization is very important to prevent pregnancy and childbirth-related complications. Therefore, this facility-based cross-sectional study aimed to provide information regarding practice of PAFP and its associated factors among women after occurrence of abortion.

More than three-fourths, 368 (77.8%) of respondents were utilized PAFP methods. This finding is in line with the studies conducted in Bahir Dar (78.5%) (15) and Gondar (74.4%) (24). However, it is lower than a study conducted in Addis Ababa (90.6%) (25). The possible reason for difference might be disparities in access to health facilities. On contrary, this finding was higher than the studies conducted in Markos (59.2%) (26) and Tigray (61.5%) (21). The possible reasons for difference might be time gap between the studies, existence of strong policy on sexual and reproductive healthcare utilization, disparities in access to health facilities, educational level, and various misconceptions about family planning methods, including rumors (27–30).

Utilization of postabortion family planning was six times more likely among 15–24 years of age groups than women with ≥45 years of age. This finding is supported by the study conducted in Gambella (31) and Bahir Dar (15). This implies that the government should give emphasis to meet the family planning need among those groups to prevent early pregnancy and childbirth-related complications (32, 33).

Also, being single and divorced were 11 and 16 times more likely to utilize PAFP methods than their counterparts. This is in fact single females and divorced are free from husband pressure, have fear of economic dependency, cultural outcast, and social discrimination once they became pregnant before marriage (34).

Women who have attended higher educational level were eight times more likely to utilize PAFP methods than their counterparts. This matched with the studies conducted in Gambella, Debre Markos, and Tigray (21, 26). As educational level of women increases, the chance of accessing information and health-seeking behavior increases, have more knowledge about reproductive health right, and could pass informed decisions about their child desire with career development put aside (35, 36). Therefore, the government should address education for all the females to use their reproductive right.

Participants with monthly income of 500–1,000 birr and >1,000 birr were eight and five times more likely to utilize PAFP methods than those who have <500 birr. This is not supported by study done in Northwest Ethiopia (37). This could be attributed to empowerment of women's and financial freedom has an impact to address reproductive health service in term of access and decision-making power and practicing their right as human being (8).

Women who did not desire current pregnancy, which end with abortion, were four times more likely to utilize PAFP methods than their counterparts. This indicates that there was unmet need of family planning before the occurrence of current pregnancy, which ends with abortion. Therefore, all the women should access all the sexual and reproductive healthcare services and they have to get the chance of self-decision about how many and when to have a child.

Respondents who had family planning counseling were nearly six times more likely to utilize PAFP methods than their counterparts. This was supported by the studies conducted in Gambella, Debre Markos, and Tigray (21, 26). This could be explained by the positive influence and the impact of counseling to client's knowledge to accept family planning and reject the rumors. This might also suggest the effectiveness of PAFP methods programs implemented in the facilities.

Women who have ever used family planning methods were four times more likely to utilize PAFP methods than their counterparts. This is in line with study conducted in Pakistan (38). This could be the fact that women who have ever used family planning methods were more familiar with side effects and rumors complaint that deter to utilize any family planning utilization. Similarly, previous users of family planning methods have knowledge and experience about its advantage than those who did not utilized.

## Conclusion

In this study, even though a significant number of participants, 368 (77.8%) of participants were using postabortion family planning service; more than two-thirds of the respondent was using only short-acting PAFP methods. Age, educational level, marital status, income, not desire of current pregnancy, counseling, and ever used family planning were factors associated with utilization of postabortion family planning. Therefore, efforts should be strengthened to improve utilization of long-acting types of PAFP to decrease the risk of unwanted pregnancy and its related complications. Further, these factors need consideration of policymakers and healthcare providers to strengthen and integrate PAFP with postabortion care package.

## Data Availability Statement

The raw data supporting the conclusions of this article will be made available by the authors, without undue reservation.

## Ethics Statement

Ethical clearance was obtained from Haramaya University, College of Health and Medical Sciences, Institutional Health Research Ethics Review Committee (HU-IHRERC). A formal letter of permission and support was written to each hospital and Health Bureau from Haramaya University. Then, the Health Bureau was wrote a cooperation letter to each health center. All the participants were informed of the purposes, procedures, risks and benefits, and the private and confidential nature of this study. More important, a written informed consent was obtained from each participant and the facility's head. Also, participants who unable to read and write gave their consent by their fingerprint after the data collectors read the consent form for them.

## Author Contributions

VM, LO, and TY contributed to the conception. AA, MY, BE, BB, MB, and BM contributed to data acquisition. VM, LO, TY, AA, MY, BE, BB, MB, and BM contributed to design, methods, analysis, and write up of the manuscript. All authors contributed to the article and approved the submitted version.

## Conflict of Interest

The authors declare that the research was conducted in the absence of any commercial or financial relationships that could be construed as a potential conflict of interest.

## Publisher's Note

All claims expressed in this article are solely those of the authors and do not necessarily represent those of their affiliated organizations, or those of the publisher, the editors and the reviewers. Any product that may be evaluated in this article, or claim that may be made by its manufacturer, is not guaranteed or endorsed by the publisher.

## References

[1] Say L Chou D. Better understanding of maternal deaths—the new WHO cause classification system. BJOG Int J Obstet Gy. (2011) 118:15. 10.1111/j.1471-0528.2011.03105.x 21951496

[2] WHO. Maternal Mortality. (2017). Avaliable online at: https://www.who.int/news-room/fact-sheets/detail/maternal-mortality (Accessed May 20, 2021).

[3] WHO. Preventing Unsafe Abortion. (2020). Available online at: https://www.who.int/news-room/fact-sheets/detail/preventing-unsafe-abortion

[4] Endalkachew Mekonnen A. Knowledge, attitude and practice (KAP) of health providers towards safe abortion provision in Addis Ababa health centers. BMC Women's Health. (2019) 19:1–10. 10.1186/s12905-019-0835-x 31727045 PMC6854666

[5] Bearak J Popinchalk A Ganatra B Moller A-B Tunçalp Ö Beavin C. Unintended pregnancy and abortion by income, region, and the legal status of abortion: estimates from a comprehensive model for 1990–2019. Lancet Globl Health. (2020) 8:e1152–e61. 10.1016/S2214-109X(20)30315-6 32710833

[6] WHO. Health Worker Roles in Providing Safe Abortion Care and Post-Abortion Contraception. (2015). Avaliable online at: https://www.ncbi.nlm.nih.gov/books/NBK316326/. (Accessed May 20, 2021).. 26401543

[7] Carolyn C H D M T. Post abortion FamilyPlanning:Addressing the Cycle Of Repeat Unintended Pregnancy and Abortion. Int Perspect Sex Reprod Health. (2010) 36:44–48.. 20403805 10.1363/ipsrh.36.044.10

[8] Sahoo J Singh SV Gupta VK Garg S Kishore J. Do socio-demographic factors still predict the choice of place of delivery: A cross-sectional study in rural North India. J Epidemiol Glob Health. (2015) 5:S27–34. 10.1016/j.jegh.2015.05.002 26073573 PMC7325830

[9] USAID. Post abortion Family Planning: Strengthening the family planning component of postabortion care. Report (2012).

[10] Janice Tripney Irene Kwan Karen Schucan . Postabortion family planning counseling and services for women in low-income countries: a systematic review. Contraception. (2013) 87:17–25. 10.1016/j.contraception.2012.07.014 22974595

[11] Opoku B. Contraceptive Preferences of post-abortion patients in Ghana. J Women's Health Care. (2012) 1:4. 10.4172/2167-0420.1000109

[12] Seid A Gebremariam A Abera M. Integration of family planning services within post abortion care at health facilities in Dessie –North East Ethiopia. J Sci Technol. (2013) 1:38. 10.4314/star.v1i1.98771

[13] Lauro D. Abortion and contraceptive use in Sub-Saharan Africa: how women plan their families. Afr J Reprod Health. (2011) 15:13–23. 21987933

[14] Abate E Smith YR Kindie W Girma A Girma Y. Prevalence and determinants of post–abortion family planning utilization in a tertiary Hospital of Northwest Ethiopia: a cross sectional study. J Contracept. (2020) 5:1–6. 10.1186/s40834-020-00143-4 33317644 PMC7737378

[15] Mekuria A Gutema H Wondiye H Abera M. Postabortion contraceptive use in Bahir Dar, Ethiopia: a cross sectional study. J Contracept. (2019) 4:1–6. 10.1186/s40834-019-0099-8 31700669 PMC6827170

[16] Lebeza A Yeshambel Agmus A Muluken A. Contraceptive use and associated factors among women seeking induced abortion in Debre Marko's town, Northwest Ethiopia: a cross-sectional study. Reprod Health. (2020) 17:97. 10.1186/s12978-020-00945-4 32552736 PMC7301557

[17] Gessessew A. Abortion and unwanted pregnancy in Adigrat Zonal Hospital, Tigray, North Ethiopia. Afr J Reprod Health. (2010) 14:183–86. 21495611

[18] CSA. Ethiopian Demographic and Health Survey 2016. Federal democratic republic of ethiopia. (2016):551.

[19] FMOH. Ethiopian Services Availability and Readiness Assessment (SARA). Ethiopian Public Health Institute (EPHI). (2018):119.

[20] Ali MH Mengistu MY Abegaz Z Bazie GW Cherie N Amsalu ET. Receiving abortion services at nongovernmental health facilities as a significant variable for postabortion family planning utilization: a comparative cross-sectional study. AJOG Global Reports. (2021) 2:100047. 10.1016/j.xagr.2021.100047 PMC9564018 36274970

[21] Moges Y Hailu T Dimtsu B Yohannes Z Kelkay B. Factors associated with uptake of post-abortion family planning in Shire town, Tigray, Ethiopia. BMC Res Notes. (2018) 11:1–6. 10.1186/s13104-018-4029-7 30591074 PMC6307259

[22] USAID. What Works: A Policy and Programe Guide to the Evidence on Post Abortion Care. Washington DC, USA: USAID (2007). p. 260.

[23] WHO. Clinical Practice Handbook for Safe Abortion. Geneva: Switzerland WHO Library Cataloguing (2014). p. 72. 24624482

[24] Dejenie Seyoum AG Gizaw Z. Assessment of post abortion contraceptive intention and associated factors among abortion clients in Gondar Town, North West Ethiopia, 2013. J Public Health. (2014) 2:215–25 10.13189/ujph.2014.020802

[25] Asrat M Bekele D Rominski SD. Post-abortion contraceptive acceptance and choice among women receiving abortion care at Saint Paul's Hospital, Addis Ababa, Ethiopia: a cross-sectional study. Lancet Glob Health. (2018) 6:S37. 10.1016/S2214-109X(18)30166-9

[26] Kokeb L Admassu E Kassa H Seyoum T. Utilization of post abortion contraceptive and associated factors among women who came for abortion service: a hospital based cross sectional study. J Fam Med Dis Prev. (2015) 1:022. 10.23937/2469-5793/1510022

[27] Sakara A Namoog MY Badu-Nyarko SK. Misconceptions and rumours about family planning among moslem males in Tamle Metropolis, Ghana. (2014) 4:9–14.

[28] Gueye A Speizer IS Corroon M Okigbo CC. Belief in family planning myths at the individual and community levels and modern contraceptive use in urban Africa. Int Perspect Sex Reprod Health. (2015) 41:191. 10.1363/intsexrephea.41.4.0191 26871727 PMC4858446

[29] Mwaisaka J Gonsalves L Thiongo M Waithaka M Sidha H Agwanda A. Exploring contraception myths and misconceptions among young men and women in Kwale County, Kenya. BMC Public Health. (2020) 20:1–10. 10.1186/s12889-020-09849-1 33176738 PMC7661170

[30] Endriyas M Eshete A Mekonnen E Misganaw T Shiferaw M. Where we should focus? Myths and misconceptions of long acting contraceptives in Southern Nations, Nationalities and People's Region, Ethiopia: qualitative study. BMC Pregnancy Childbirth. (2018) 18:1–6. 10.1186/s12884-018-1731-3 29653581 PMC5899320

[31] Abdulhakim, Abamecha, Alemayehu, Shiferaw, Ashenafi, Kassaye. Assessment of post abortion contraceptive intention and associated factors among abortion clients in Gambella Health Facilities, Gambella Town, South West Ethiopia. Valley Int J. (2016) 3:10.

[32] WHO. Contraceptions. (2017). Avaliable online at: https://www.who.int/health-topics/contraception. (Accessed May 20, 2021)..

[33] Ahmed O. 13 Ways States Can Protect Advance Women's Health Rights. (2021). Avaliable online at: https://wwwamericanprogressorg/issues/women/reports/2018/11/30/461639/13-ways-states-can-protect-advance-womens-health-rights/ (Accessed November 30, 2018).

[34] Birhanu BE Kebede DL Kahsay AB Belachew AB. Predictors of teenage pregnancy in Ethiopia: a multilevel analysis. BMC Public Health. (2019) 19:1–10. 10.1186/s12889-019-6845-7 31101101 PMC6525551

[35] Zhou Y Wang T Fu J Chen M Meng Y Luo Y. Access to reproductive health services among the female floating population of childbearing age: a cross-sectional study in Changsha, China. BMC Health Serv Res. (2019) 19:1–10. 10.1186/s12913-019-4334-4 31370834 PMC6676621

[36] Heera K Shrestha M Pokharel N Niraula SR Pyakurel P Parajuli SB. Women's empowerment for abortion and family planning decision making among marginalized women in Nepal: a mixed method study. Reprod Health. (2021) 18:1–11. 10.1186/s12978-021-01087-x 33541377 PMC7863411

[37] Ayele, Hamba, Gudeta. Assessment of the prevalence of unplanned pregnancy and associated factors among pregnant women attending antenatal care unit at Hambiso Health Center Hambiso, North Shewa, Ethiopia. J Women's Health Care. (2017) 6:5.

[38] Shah IH Santhya KG Cleland J. Postpartum and Post-Abortion Contraception: From Research to Programs. Stud Fam Plann. (2015) 46:343–53. 10.1111/j.1728-4465.2015.00036.x 26643486

